# Targeting the MUC1-C oncoprotein inhibits self-renewal capacity of breast cancer cells

**DOI:** 10.18632/oncotarget.1848

**Published:** 2014-03-24

**Authors:** Maroof Alam, Hasan Rajabi, Rehan Ahmad, Caining Jin, Donald Kufe

**Affiliations:** ^1^ Dana-Farber Cancer Institute Harvard Medical School Boston, MA; ^2^ Present address: College of Medicine, King Saud University, Riyadh, Saudi Arabia; ^3^ Present address: Genus Oncology, Boston, MA

**Keywords:** MUC1, breast cancer, stem-like cells, mammospheres, tumorigenicity, NF-κB

## Abstract

The capacity of breast cancer cells to form mammospheres in non-adherent serum-free culture is used as a functional characteristic of the self-renewing stem-like cell population. The present studies demonstrate that silencing expression of the MUC1-C oncoprotein inhibits growth of luminal MCF-7 and HER2-overexpressing SKBR3 breast cancer cells as mammospheres. We also show that triple-negative MDA-MB-468 breast cancer cells are dependent on MUC1-C for growth as mammospheres and tumor xenografts. Similar results were obtained when MUC1-C function was inhibited by expression of a MUC1-C(CQC→AQA) mutant. Moreover, treatment with the MUC1-C inhibitor GO-203, a cell penetrating peptide that binds to the MUC1-C cytoplasmic domain and blocks MUC1-C function, confirmed the importance of this target for self-renewal. The mechanistic basis for these findings is supported by the demonstration that MUC1-C activates NF-κB, occupies the IL-8 promoter with NF-κB, and induces IL-8 transcription. MUC1-C also induces NF-κB-dependent expression of the IL-8 receptor, CXCR1. In concert with these results, targeting MUC1-C with GO-203 suppresses IL-8/CXCR1 expression and disrupts the formation of established mammospheres. Our findings indicate that MUC1-C contributes to the self-renewal of breast cancer cells by activating the NF-κB→IL-8/CXCR1 pathway and that targeting MUC1-C represents a potential approach for the treatment of this population.

## INTRODUCTION

Normal tissues contain stem cell populations that renew through asymmetrical division and give rise to progenitors committed to functional differentiation [1]. Cancer stem-like cells are similarly capable of self-renewal and have the capacity for generating diverse progeny that comprise the tumor [1, 2]. Stem-like cells from hematologic malignancies and solid tumors have thus been defined in part by their self-renewal and tumor-initiating potential [3]. Nonetheless, debate remains about the definition of cancer stem-like cells as a result of their heterogeneity and plasticity [4, 5]. In this context, cell surface markers, such as CD44 and CD133, have been used to isolate subsets enriched for stem-like cells in solid tumors [5]. In addition, aldehyde dehydrogenase (ALDH) activity has been a focus of study as a marker for both normal and cancer stem cells [6]. Indeed, many of these markers are not exclusively expressed by cancer stem-like cells and the available evidence indicates that there is considerable phenotypic heterogeneity within these populations [4, 5]. With regard to functional characteristics, a hallmark of cancer stem-like cells is their relative resistance to apoptosis in the response to genotoxic stress induced by anti-cancer drugs and radiation [7]. Moreover, cancer stem-like cells have been characterized by their ability to undergo the epithelial-mesenchymal transition (EMT), a process that endows more differentiated epithelial cells with stem cell characteristics [8]. The induction of EMT results in both the acquisition of mesenchymal traits necessary for invasion and metastases, and the expression of CSC markers [8]. EMT also increases the capacity of mammary epithelial cells to form mammospheres in non-adherent serum-free culture [8], a characteristic that is dependent on the presence of self-renewing stem cells [9, 10]. The capacity for mammosphere formation has thus emerged as another hallmark of the breast cancer stem-like cell [9]. From a mechanistic standpoint, NF-κB signaling has been linked to self-renewal, mammosphere formation and breast cancer-initiating cells [11-13]. In addition, interleukin-8 (IL-8), an inflammatory cytokine that is upregulated in breast cancer and is associated with a poor prognosis [14], has been identified as an important regulator of EMT, stem-like cell activity and mammosphere formation [15-18].

Mucin 1 (MUC1) is a heterodimeric transmembrane protein that is aberrantly overexpressed in human breast cancers as a result in part of *MUC1* gene amplification and dysregulation of its transcription [19]. The functional role of MUC1 in tumorigenesis was advanced by the finding that MUC1 undergoes autocleavage into two subunits, which in turn form a stable non-covalent heterodimer [19]. The extracellular N-terminal subunit (MUC1-N) is the mucin component of the heterodimer and is tethered to the cell surface in a complex with the transmembrane C-terminal subunit (MUC1-C) [19]. MUC1-C consists of a 58-amino acid (aa) extracellular domain, a transmembrane region and a 72-aa cytoplasmic tail [19]. MUC1-C interacts with receptor tyrosine kinases (RTKs), such as EGFR and HER2, at the cell membrane and contributes to their activation [19, 20]. In this way, targeting MUC1-C with silencing downregulates p-HER2 activation in HER2-overexpressing breast cancer cells [20]. Moreover, inhibition of MUC1-C with GO-203, a cell penetrating peptide that binds to the MUC1-C cytoplasmic domain at the CQC motif and blocks MUC1-C function [21, 22], suppresses p-HER2 activation [20]. MUC1-C has also been linked to regulation of downstream RTK signaling, such as the PI3K→AKT and MEK→ERK pathways [19, 20, 23]. In addition, MUC1-C is imported into the nucleus by importin-β, where it interacts with transcription factors and contributes to their transactivating function [19, 24]. In this regard, MUC1-C associates with NF-κB p65 and induces activation of the *ZEB1* gene by a NF-κB-mediated mechanism [25]. In turn, ZEB1 suppresses miR-200c expression and thereby induces EMT and cellular invasion by a MUC1-C-mediated mechanism [25]. In addition, recent studies have shown that MUC1-C interacts with the CCAAT/enhancer-binding protein β (C/EBPβ) on the *ALDH1A1* gene promoter and induces C/EBPβ-mediated ALDH1A1 expression [23]. The available evidence thus links MUC1-C to the induction of EMT [25] and ALDH activity [23], both characteristics of breast cancer stem-like cell populations. Other studies of breast cancer cells have demonstrated that MUC1 is detectable in “side populations” that express the ABCG2 transporter, which has been used as marker of stem/progenitor cells [26]. Overexpression of MUC1, as found in breast cancer cells, is also associated with resistance to apoptosis in response to genotoxic anti-cancer agents [27]. One study has demonstrated that MUC1 expression is increased in breast cancer cells that form mammospheres [28]; whereas, another publication reported that MUC1 is decreased under these conditions of anchorage-independent growth [29]. Of relevance to the present work, there is no available information that addresses whether MUC1-C is involved in mammosphere formation or in activation of the IL-8 pathway that contributes to the growth of breast cancer cells as spheres.

The present studies demonstrate that MUC1-C is upregulated under nonadherent culture conditions, which select for self-renewing breast cancer cells. The results further demonstrate that silencing MUC1-C blocks the capacity of luminal, HER2-overexpressing and triple-negative breast cancer cells to form mammospheres. Targeting MUC1-C homodimerization by expression of a MUC1-C(CQC→AQA) mutant or the MUC1-C inhibitor GO-203 also blocks self-renewal of breast cancer cells. The mechanistic basis for these results is supported by the demonstration that MUC1-C activates NF-κB and thereby expression of IL-8 and CXCR1. Our findings indicate that targeting MUC1-C represents an approach to inhibit the self-renewal capacity of breast cancer cells.

## RESULTS

### MUC1-C expression is upregulated in MCF-7 cell mammospheres

**Figure 1:**
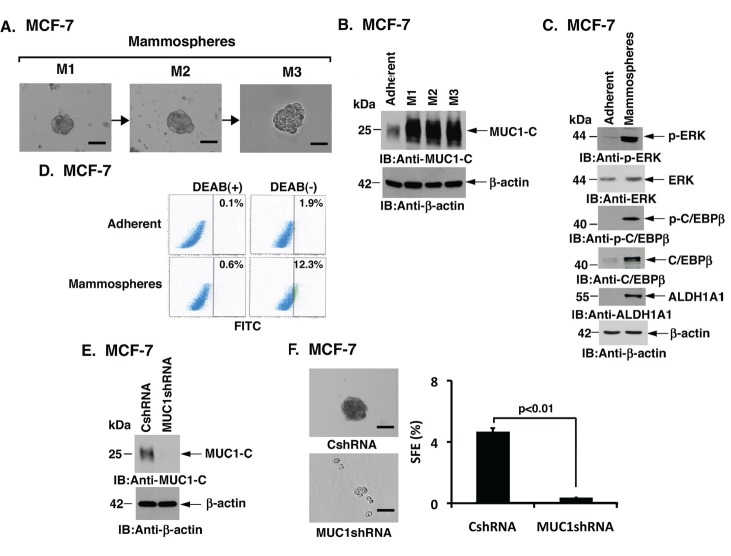
Silencing MUC1 expression attenuates formation of MCF-7 cell mammospheres A. Representative phase contrast microscopy images are shown for MCF-7 mammospheres that were serially passaged for three generations (M1 to M3). Bar represents 50 microns. B. Lysates of MCF-7 cells cultured as an adherent monolayer or as M1, M2 and M3 mammospheres were immunoblotted with the indicated antibodies. C. Lysates of MCF-7 cells cultured as an adherent monolayer or as mammospheres were immunoblotted with the indicated antibodies. D. MCF-7 cells grown as monolayers and as mammospheres were incubated with the ALDH substrate (BAAA) and the ALDH1 inhibitor (DEAB). The percentage of aldefluor-positive cells is included in the panels. E. MCF-7 cells were infected with lentiviruses to stably express a control scrambled shRNA (CshRNA) or a MUC1 shRNA. Lysates from the MCF-7/CshRNA and MCF-7/MUC1shRNA cells were immunoblotted with the indicated antibodies. F. Images are shown for MCF-7/CshRNA and MCF-7/MUC1shRNA cells grown as mammospheres (left). Bar represents 50 microns. The percentage sphere forming efficiency (SFE) is expressed as the mean±SD of three determinations (right).

To assess the potential involvement of MUC1-C in conferring anchorage-independent growth, luminal ER+ MCF-7 breast cancer cells were established as mammospheres and serially passaged for three generations (M1 to M3) (Fig. 1A). MUC1-C expression was found to be substantially upregulated in M1, M2 and M3 mammospheres as compared to that in MCF-7 cells grown as an adherent monolayer (Fig. 1B). In concert with the demonstration that MUC1-C activates ERK→C/EBPβ signaling and induction of ALDH1A1 [23], we found activation of this pathway in MCF-7 mammospheres as evidenced by increased p-ERK, p-C/EBPβ and ALDH1A1 levels (Fig. 1C). In addition, the upregulation of MUC1-C expression in MCF-7 mammospheres was associated with increases in aldefluor activity (Fig. 1D). Based on these findings, we silenced MUC1-C in MCF-7 cells to assess its functional role in sphere formation (Fig. 1E). Notably, MUC1-C silencing resulted in a significant reduction in mammosphere formation as indicated by marked decreases in both sphere size (Fig. 1F, left) and sphere forming efficiency (SFE) (Fig. 1F, right), indicating that MUC1-C is of functional importance for growth of breast cancer cells under anchorage-independent conditions.

### MUC1-C confers MCF-7 mammosphere formation

**Figure 2:**
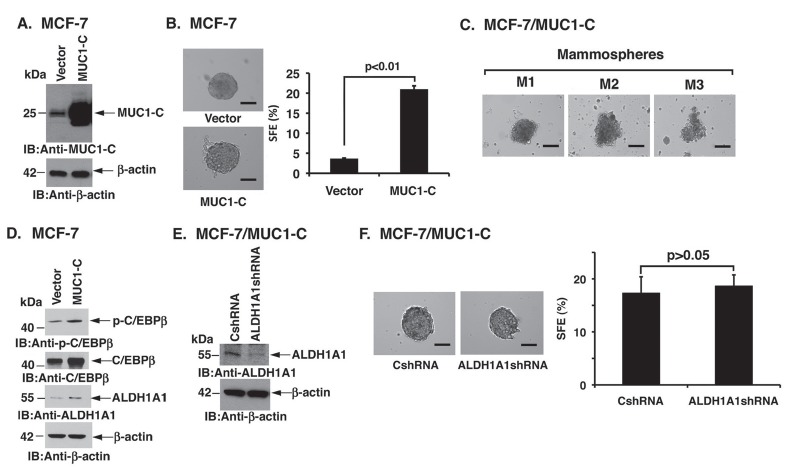
Overexpression of MUC1-C induces MCF-7 mammosphere formation A. MCF-7 cells were infected with lentiviruses to stably express a control vector or MUC1-C. Lysates from the MCF-7/vector and MCF-7/MUC1-C cells were immunoblotted with the indicated antibodies. B. Representative images are shown for MCF-7/vector and MCF-7/MUC1-C mammospheres (left). The percentage SFE is expressed as the mean±SD of three determinations (right). Bar represents 50 microns. C. Representative images are shown for MCF-7/MUC1-C cells grown as M1, M2 and M3 mammospheres. Bar represents 50 microns. D. Lysates of MCF-7/vector and MCF-7/MUC1-C cells grown as mammospheres were immunoblotted with the indicated antibodies. E. MCF-7/MUC1-C cells were infected with lentiviruses that stably express a control scrambled shRNA (CshRNA) or an ALDH1A1 shRNA. Lysates from MCF-7/ MUC1-C/ CshRNA and MCF-7/MUC1-C/ALDH1A1shRNA cells were immunoblotted with the indicated antibodies. F. Representative images are shown for MCF-7/MUC1-C/CshRNA and MCF-7/MUC1-C/ALDH1A1shRNA cells grown as mammospheres (left). Bar represents 50 microns. The percentage SFE is expressed as the mean±SD of three determinations (right).

To further address the notion that MUC1-C regulates mammosphere formation, we generated MCF-7 cells that stably overexpress the MUC1-C subunit (Fig. 2A). MUC1-C overexpression had little effect on M1 mammosphere size (Fig. 2B, left), but significantly increased SFE from 3-4% to over 15% (Fig. 2B, right). MCF-7/MUC1-C cells also generated M2 and M3 mammospheres that were somewhat more diffuse (Fig. 2C) than that observed with MCF-7 cells (Fig. 1A), a finding that has been attributed to the development of EMT characteristics [30]. Adherent MCF-7/MUC1-C cells exhibit upregulation of the ERK→C/EBPβ→ALDH1A1 pathway [23]. Similar findings were observed in the MCF-7/MUC1-C cells grown as mammospheres as supported by increased p-C/EBPβ and ALDH1A1 levels (Fig. 2D). To determine whether MUC1-C-induced ALDH1 activity contributes to mammosphere formation, we silenced ALDH1A1 in MCF-7/MUC1-C cells (Fig. 2E). Of note, downregulation of ALDH1A1 expression had no apparent effect on mammosphere size (Fig. 2F, left) or SFE (Fig. 2F, right), indicating that MUC1-C confers non-adherent growth by an ALDH1A1-independent mechanism.

### HER2-overexpressing and triple-negative breast cancer cells are dependent on MUC1-C for mammosphere formation

**Figure 3:**
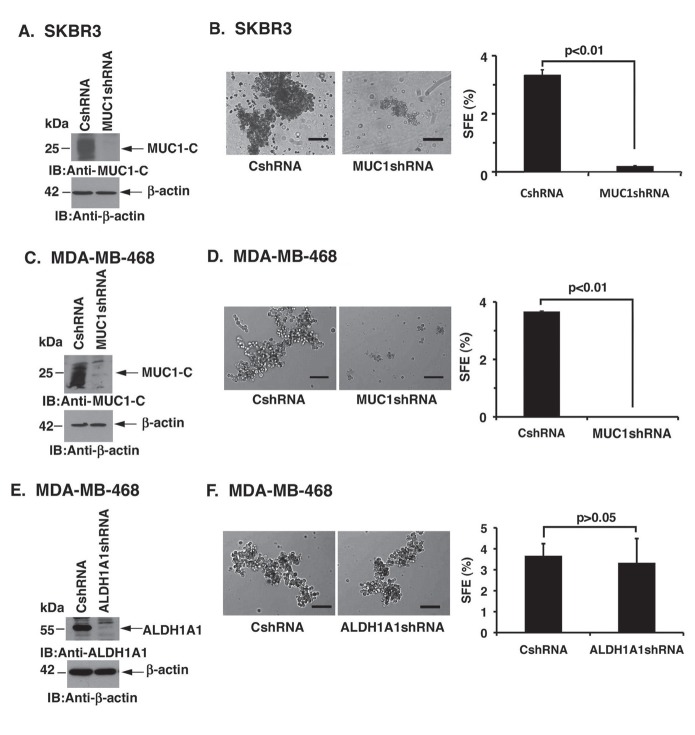
HER2-overexpressing and triple-negative breast cancer cells are dependent on MUC1-C for mammosphere formation A.SKBR3 cells were infected with lentiviruses stably expressing a CshRNA or MUC1shRNA. Lysates from SKBR3/CshRNA and SKBR3/MUC1shRNA cells were immunoblotted with the indicated antibodies. B. Representative images are shown of SKBR3/CshRNA and SKBR3/MUC1shRNA cells grown in mammosphere culture medium (left). Bar represents 100 microns. The percentage SFE is expressed as the mean±SD of three determinations (right). C. MDA-MB-468 cells were infected with lentiviruses stably expressing CshRNA or MUC1shRNA. Lysates of MDA-MB-468/CshRNA and MDA-MB-468/MUC1shRNA cells were immunoblotted with the indicated antibodies. D. Representative images are shown of MDA-MB-468/CshRNA and MDA-MB-468/MUC1shRNA cells grown in mammosphere culture medium (left). Bar represents 100 microns. The percentage SFE is expressed as the mean±SD of three determinations (right). E. MDA-MB-468 cells were infected with lentiviruses that stably express a control CshRNA or an ALDH1A1 shRNA. Lysates were immunoblotted with the indicated antibodies. F. Representative images are shown of MDA-MB-468/CshRNA and MDA-MB-468/ALDH1A1shRNA cells grown as mammospheres (left). Bar represents 100 microns. The percentage SFE is expressed as the mean±SD of three determinations (right).

To extend these findings with luminal MCF-7 cells to other breast cancer cell types, we first studied HER2-overexpressing SKBR3 cells. As found for MCF-7 cells, stable silencing of MUC1-C in SKBR3 cells (Fig. 3A) was associated with a marked decrease in mammosphere size (Fig. 3B, left) and SFE (Fig. 3B, right). Silencing MUC1-C in triple-negative (ER-/PR-/HER2-) MDA-MB-468 cells (Fig. 3C) also resulted in substantial suppression of mammosphere size (Fig. 3D, left) and SFE (Fig. 3D, right), indicating that these effects of silencing MUC1-C are independent of breast cancer cell subtype. Similar results were obtained when MUC1-C was downregulated in MDA-MB-468 cells using a different shRNA (Supplemental Figs. S1A and B), further indicating that the findings are not the result of shRNA off-target effects. Silencing MUC1-C in MDA-MB-468 cells causes downregulation of the ERK→C/EBPβ→ALDH1 pathway and loss of ALDH1 activity [23]. We therefore silenced ALDH1A1 in MDA-MB-468 cells to assess their dependence on ALDH1A1 for sphere formation (Fig. 3E). As found for MCF-7 cells, the results demonstrate that MDA-MB-468 mammosphere formation is conferred by a MUC1-C-dependent, ALDH1A1-independent mechanism (Fig. 3F, left and right).

### Targeting MUC1-C homodimerization abrogates mammosphere formation

**Figure 4:**
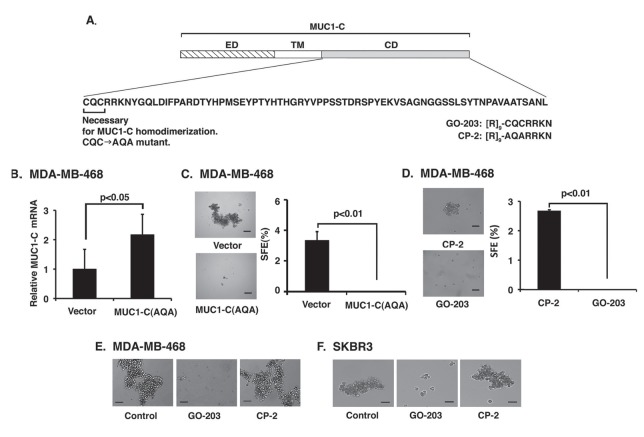
Targeting MUC1-C homodimerization suppresses mammosphere formation A. Schema of the MUC1-C subunit (ED, extracellular domain; TM, transmembrane region) and the amino acid (aa) sequence of the cytoplasmic domain (CD). Highlighted is the CQC motif that is necessary for MUC1-C homodimerization. CQC has been mutated to AQA in the MUC1-C(AQA) vector. The CQC motif is the target of the GO-203 peptide. The control CP-2 peptide differs from GO-203 in that the critical CQC motif has been altered to AQA. B. MDA-MB-468 cells expressing a control vector or a MUC1-C(CQC→AQA) mutant were analyzed for MUC1-C mRNA levels by qRT-PCR. The results are expressed as relative MUC1-C mRNA levels (mean±SD of three determinations) as compared with that obtained for GAPDH (vector cells assigned a value of 1). C. Representative images are shown of MDA-MB-468/vector and MDA-MB-468/MUC1-C(AQA) cells grown in mammosphere culture medium (left). Bar represents 100 microns. The percentage SFE is expressed as the mean±SD of three determinations (right). D. MDA-MB-468 cells were suspended in mammosphere culture medium containing 5 μM CP-2 or 5 μM GO-203. Images taken after 48 h are shown for the CP-2- and GO-203-treated cells (left). Bar represents 100 microns. The percentage SFE is expressed as the mean±SD of three determinations (right). E. MDA-MB-468 cells were established as mammospheres (left) and then treated with 5 μM GO-203 (middle) or 5 μM CP-2 (right) for 36 h. Bar represents 100 microns. F. SKBR3 cells were established as mammospheres (left) and then treated with 5 μM GO-203 (middle) or 5 μM CP-2 (right) for 24 h. Bar represents 100 microns.

The MUC1-C cytoplasmic domain contains a CQC motif that is necessary for the formation of MUC1-C homodimers and thereby the MUC1-C oncogenic function (Fig. 4A) [19]. To search for further evidence linking MUC1-C to mammosphere formation, we stably expressed a MUC1-C(CQC→AQA) mutant in MDA-MB-468 cells (Fig. 4B). MUC1-C(CQC→AQA) expression was associated with marked suppression of MDA-MB-468 mammosphere formation (Fig. 4C), indicating that MUC1-C homodimerization is necessary for conferring anchorage-independent growth. Previous work showed that MUC1-C homodimerization is inhibited by the cell-penetrating peptide, GO-203, which contains a poly-Arg transduction domain linked to CQCRRKN and binds to endogenous MUC1-C at the CQC motif [21](Fig. 4A). Notably, GO-203 treatment blocked the formation of MDA-MB-468 mammospheres (Fig. 4D). By contrast, the CP-2 peptide, which includes AQARRKN and is not active in targeting the MUC1-C CQC motif (Fig. 4A) [21], had no apparent effect on sphere formation (Fig. 4D, left and right). GO-203, but not CP-2, treatment for 36 h was also highly effective in disrupting established MDA-MB-468 mammospheres (Fig. 4E). Moreover, established SKBR3 mammospheres were disrupted by targeting MUC1-C with GO-203 (Fig. 4F), providing further evidence that supports the importance of MUC1-C in conferring anchorage-independent growth and self-renewal.

### MUC1-C induces mammosphere formation by an NF-κB-dependent mechanism

**Figure 5:**
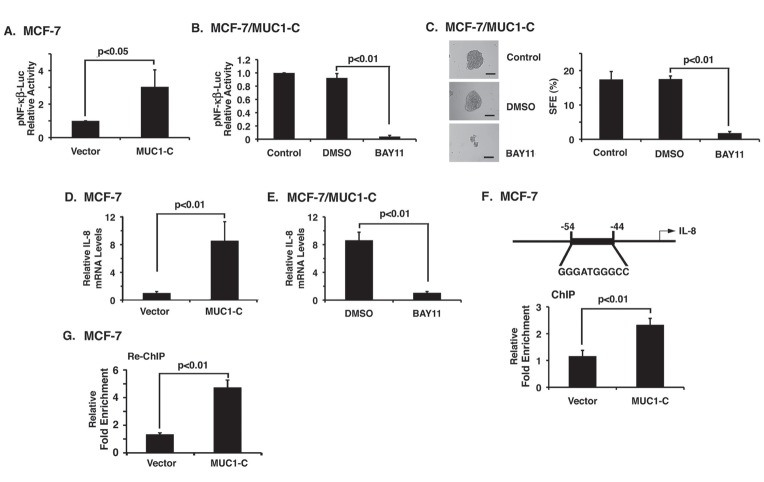
MUC1-C induces mammosphere formation by an NF-κB-dependent mechanism A. MCF-7/vector and MCF-7/MUC1-C cells were cotransfected with p-NF-κB/Luc and control pRL-TK plasmids. Luciferase activity was measured 48 h after transfection. Results are expressed as relative luciferase activity (mean±SD of three determinations) compared to that obtained with MCF-7/vector cells (assigned a value of 1). B. MCF-7/MUC1-C cells were cotransfected with the p-NF-κB/Luc and pRL-TK plasmids. After 48 h, the transfected cells were left untreated (Control) and treated with DMSO as vehicle or with 5 μM BAY11-7085 for an additional 12 h. Results are expressed as relative luciferase activity (mean±SD of three determinations) as compared to that obtained for the control untreated cells (assigned a value of 1). C. MCF-7/MUC1-C cells were suspended in mammosphere culture medium and left untreated (Control) or treated with DMSO or 5 μM BAY11-7085. Cells were imaged after 5 days (left). Bar represents 50 microns. The percentage SFE is expressed as the mean±SD of three determinations (right). D and E. MCF-7/vector and MCF-7/MUC1-C cells were analyzed for IL-8 mRNA levels by qRT-PCR (vector cells assigned a value of 1). (D). MCF-7/MUC1-C cells were treated with DMSO or 5 μM BAY11-7085 for 12 h (E). The results are expressed as relative IL-8 mRNA levels (mean±SD of three determinations) as compared with that obtained for GAPDH (BAY11-treated cells assigned a value of 1). F. Schematic representation of the IL-8 promoter with positioning of the NF-κB site. Soluble chromatin from MCF-7/vector and MCF-7/MUC1-C cells was precipitated with anti-NF-κB or a control IgG. The final DNA samples were amplified by qPCR with pairs of primers for the NF-κB binding region (NBR; −117 to −29) or a control region (CR; −4840 to −4775). The results (mean±SD of three determinations) are expressed as the relative fold enrichment compared with that obtained with the IgG control (vector cells assigned a value of 1). G. Soluble chromatin from MCF-7/vector and MCF-7/MUC1-C cells was precipitated with anti-NF-κB, released and reimmunoprecipitated with anti-MUC1-C. The final DNA samples were amplified by qPCR with pairs of primers for the IL-8 promoter NBR and CR. The results (mean±SD of three determinations) are expressed as the relative fold enrichment compared with that obtained with the IgG control (vector cells assigned a value of 1).

Activation of the NF-κB pathway has been linked to breast cancer-initiating cell activity [11-13]. Other studies have shown that aberrant expression of MUC1-C activates NF-κB signaling [24, 31]. Therefore, to determine whether MUC1-C confers mammosphere formation through induction of NF-κB, we first asked if overexpression of MUC1-C in MCF-7 cells is associated with an increase in NF-κB activity. Using an NF-κB-driven promoter-reporter, we found a significant increase in NF-κB activity in MCF-7/MUC1-C cells as compared to that obtained in MCF-7/vector cells (Fig. 5A). Treatment of MCF-7/MUC1-C cells with the NF-κB inhibitor BAY11-7085 was associated with suppression of NF-κB activity (Fig. 5B). Moreover, MUC1-C-induced MCF-7 mammosphere formation was inhibited by BAY11-7085 (Figs. 5C, left and right), indicating that MUC1-C induces NF-κB activity and thereby anchorage-independent growth. With regard to downstream signaling, NF-κB has been shown to confer stemness and the formation of spheres by upregulating expression of IL-8 [32, 33]. In this context, we observed marked increases in IL-8 mRNA levels in MCF-7/MUC1-C, as compared to MCF-7/vector, cells (Fig. 5D) that was suppressed in MCF-7/MUC1-C cells by inhibition of NF-κB with BAY11-7085 (Fig. 5E). MUC1-C binds directly to NF-κB p65 and promotes recruitment of MUC1-C/NF-κB complexes to the promoters of NF-κB target genes [24]. By extension, ChIP analysis of the IL-8 promoter, which contains an NF-κB binding site, demonstrated that NF-κB p65 occupancy is increased in MCF-7/MUC1-C cells (Fig. 5F) and that, in re-ChIP studies, MUC1-C is detectable on the IL-8 promoter with NF-κB p65 (Fig. 5G). These findings provided support for a mechanism in which MUC1-C activates NF-κB and thereby induces IL-8 expression and mammosphere formation.

### Silencing MUC1-C suppresses IL-8 mRNA levels

**Figure 6:**
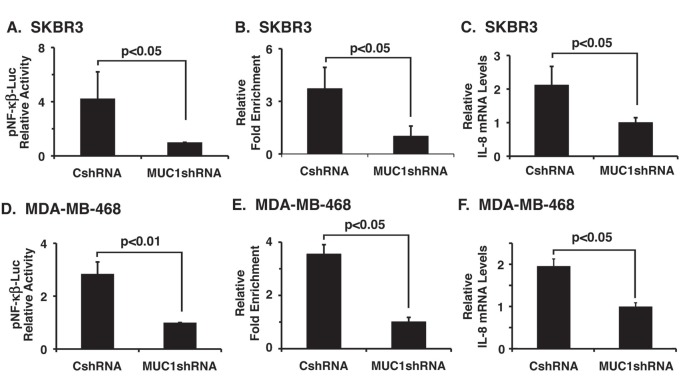
Silencing MUC1-C in SKBR3 and MDA-MB-468 cells suppresses IL-8 expression A. SKBR3/CshRNA and SKBR3/MUC1shRNA cells were cotransfected with p-NF-κB/Luc and control pRL-TK plasmids. Luciferase activity was measured 48 h after transfection. Results are expressed as relative luciferase activity (mean±SD of three determinations) compared to that obtained with SKBR3/MUC1shRNA cells (assigned a value of 1). B. Soluble chromatin from SKBR3/CshRNA and SKBR3/MUC1shRNA cells was precipitated with anti-NF-κB or a control IgG. The final DNA samples were amplified by qPCR with pairs of primers for the NBR or CR. The results (mean±SD of three determinations) are expressed as the relative fold enrichment compared with that obtained with the IgG control (MUC1shRNA cells assigned a value of 1). C. SKBR3/CshRNA and SKBR3/MUC1shRNA cells were analyzed for IL-8 mRNA levels. The results are expressed as relative IL-8 mRNA levels (mean±SD of three determinations) as compared with that obtained for GAPDH (MUC1shRNA cells assigned a value of 1). D. MDA-MB-468/CshRNA and MDA-MB-468/MUC1shRNA cells were cotransfected with p-NF-κB/Luc and pRL-TK plasmids. Luciferase activity was measured 48 h after transfection. Results are expressed as relative luciferase activity (mean±SD of three determinations) compared to that obtained with MDA-MB-468/MUC1shRNA cells (assigned a value of 1). E. Soluble chromatin from MDA-MB-468/CshRNA and MDA-MB-468/MUC1shRNA cells was precipitated with anti-NF-κB or a control IgG. The final DNA samples were amplified by qPCR with pairs of primers for the NBR or CR. The results (mean±SD of three determinations) are expressed as the relative fold enrichment compared with that obtained with the IgG control (MUC1shRNA cells assigned a value of 1). F. MDA-MB-468/CshRNA and MDA-MB-468/MUC1shRNA cells were analyzed for IL-8 mRNA levels. The results are expressed as relative IL-8 mRNA levels (mean±SD of three determinations) as compared with that obtained for GAPDH (MUC1shRNA cells assigned a value of 1).

Activation of HER2 in breast cancer cells promotes stemness and sphere formation by upregulation of NF-κB and IL-8 signaling [34, 35]. Moreover, IL-8 plays a critical role in the acquisition and maintenance of EMT [17]. Our observation that silencing MUC1-C in SKBR3 cells blocks mammosphere formation (Figs. 3B and C) thus prompted studies to evaluate NF-κB activation and IL-8 expression in this model. As compared to SKBR3/CshRNA cells, downregulation of MUC1-C in SKBR3/MUC1shRNA cells was associated with suppression of NF-κB activity (Fig. 6A) and NF-B occupancy on the IL-8 promoter (Fig. 6B). Silencing MUC1-C in SKBR3 cells was also associated with decreases in IL-8 mRNA levels (Fig. 6C). To extend this line of investigation to MDA-MB-468 cells, we found that silencing MUC1-C similarly suppresses NF-κB activity (Fig. 6D), NF-κB occupancy on the IL-8 promoter (Fig. 6E) and IL-8 mRNA levels (Fig. 6F). We also found that MDA-MB-468 cells stably silenced for NF-κB p65 (Supplemental Fig. S2A) exhibit decreases in IL-8 expression (Supplemental Fig. S2B). Moreover, as shown for MDA-MB-468/MUC1shRNA cells, silencing NF-κB p65 was associated with suppression of mammosphere formation (Supplemental Fig. S2C, left and right). These findings thus provided further support for activation of a MUC1-C→NF-κB→IL-8 pathway.

**Figure 7:**
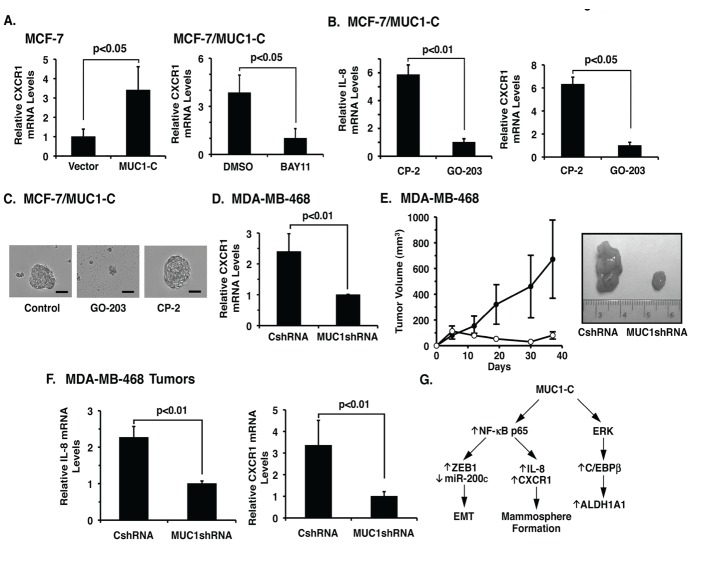
Targeting MUC1-C downregulates IL-8 and CXCR1 expression and blocks tumorigenicity A. MCF-7/vector and MCF-7/MUC1-C cells were analyzed for CXCR1 mRNA levels by qRT-PCR (left). MCF-7/MUC1-C cells were treated with DMSO or 5 μM BAY11-7085 for 12 h (right). The results are expressed as relative CXCR1 mRNA levels (mean±SD of three determinations) as compared with that obtained for GAPDH (vector cells and BAY11-treated cells assigned a value of 1). B. MCF-7/MUC1-C cells were treated with 5 μM CP-2 or GO-203 for 48 h and then analyzed for IL-8 (left) and CXCR1 (right) mRNA levels by qRT-PCR. The results are expressed as relative CXCR1 mRNA levels (mean±SD of three determinations) as compared with that obtained for GAPDH (GO-203-treated cells assigned a value of 1). C. MCF-7/MUC1-C cells were established as mammospheres (left) and then treated with 5 μM GO-203 (middle) or 5 μM CP-2 (right) for 48 h. Bar represents 50 microns. D. MDA-MB-468/CshRNA and MDA-MB-468/MUC1shRNA cells were analyzed for CXCR1 mRNA levels. The results are expressed as relative CXCR1 mRNA levels (mean±SD of three determinations) as compared with that obtained for GAPDH (MUC1shRNA cells assigned a value of 1). E. MDA-MB-468/CshRNA (closed circles) and MDA-MB-468/MUC1shRNA (open circles) cells were injected subcutaneously in the flanks of female nude mice. Tumor volumes were determined on the indicated days after injection. The results are expressed as tumor volumes (mean±SEM for 3 mice) (left). Representative MDA-MB-468/CshRNA and MDA-MB-468/MUC1shRNA tumors were excised on day 37 (right). F. MDA-MB-468/CshRNA and MDA-MB-468/MUC1shRNA tumor cells were analyzed for IL-8 (left) and CXCR1 (right) mRNA levels by qRT-PCR. The results are expressed as relative mRNA levels (mean±SD of three determinations) as compared with that obtained for GAPDH (MUC1shRNA cells assigned a value of 1). G. Schema depicting MUC1-C-induced activation of NF-κB p65 and thereby induction of IL-8/CXCR1 expression and mammosphere formation. Targeting MUC1-C with (i) silencing, (ii) expressing a MUC1-C(CQC→AQA) mutant, or (iii) GO-203 treatment suppresses NF-κB activity, decreases IL-8/CXCR1 expression and inhibits mammosphere formation. MUC1-C also activates (i) NF-κB p65-mediated induction of ZEB1 and EMT [25], and (ii) ERK→C/EBPβ signaling and ALDH1A1 expression [23]. Our results demonstrate that silencing ALDH1A1 has no detectable effect on mammosphere formation, indicating that the MUC1-C→NF-κB and MUC1-C→ALDH1A1 pathways confer distinct functions.

Targeting MUC1-C downregulates both IL-8 and CXCR1 expression. Other work has shown that NF-κB induces expression of the IL-8 receptor, CXCR1 [36]. In this regard and like IL-8, CXCR1 expression was increased in MCF-7/MUC1-C cells (Fig. 7A, left). Additionally, MUC1-C-induced increases in CXCR1 expression were inhibited by BAY11-7085 (Fig. 7A, right), supporting an NF-κB-mediated mechanism. Blocking MUC1-C homodimerization with GO-203 disrupts the interaction with NF-κB p65 and activation of the NF-κB pathway [24]. In this regard, treatment of MCF-7/MUC1-C cells with GO-203 was associated with downregulation of both IL-8 (Fig. 7B, left) and CXCR1 (Fig. 7B, right). GO-203, but not CP-2, treatment was also associated with disruption of established MCF-7/MUC1-C mammospheres (Fig. 7C). In concert with these findings and the downregulation of IL-8 (Fig. 6F), silencing MUC1-C in MDA-MB-468 cells was associated with decreases in CXCR1 mRNA levels (Fig. 7D). Similar results were obtained in MDA-MB-468 cells silenced for NF-κB p65 (Supplemental Fig. S3), confirming the induction of CXCR1 expression by an NF-κB-dependent mechanism. To extend these observations, we investigated the effects of silencing MUC1-C on tumorigenicity of MDA-MB-468 cells. In concert with a decrease in the capacity for mammosphere formation, growth of MDA-MB-468/MUC1shRNA cells as tumor xenografts was substantially inhibited as compared to that obtained for MDA-MB-468/CshRNA cells (Fig. 7E, left and right). qRT-PCR analysis of the MDA-MB-468 cells growing as tumors further demonstrated that silencing MUC1-C results in decreased expression of IL-8 (Fig. 7F, left) and CXCR1 (Fig. 7F, right). These findings collectively indicated that targeting MUC1-C suppresses NF-κB→IL-8/CXCR1 signaling and thereby mammosphere formation and tumorigenicity.

## DISCUSSION

MUC1 is aberrantly overexpressed in luminal, HER2+ and basal-like triple-negative breast cancers [19, 37] as a result in part of *MUC1* gene alterations and dysregulation of transcription [19]. The MUC1-C subunit has also been linked to ER function [38-40] and HER2 activation [20] in breast cancer cells. Strikingly, however, little is known about whether MUC1-C is of importance for the growth and survival of breast cancer cells that have the capacity for self-renewal. The present studies demonstrate that growth of luminal MCF-7 breast cancer cells as mammospheres, which enriches for self-renewing breast cancer cells that survive anoikis under nonadherent conditions [9, 10], is associated with substantial upregulation of MUC1-C expression. Consistent with a potential functional role, silencing MUC1-C in MCF-7 cells decreased mammosphere formation. In addition, overexpression of MUC1-C in this model increased sphere forming efficiency. Anchorage-independent growth is associated with induction of ALDH1 activity, supporting ALDH1 as a marker of human mammary stem cells [41]. Additionally, recent studies in MCF-7 and other breast cancer cells have shown that MUC1-C increases ALDH1A1 expression [23], raising the possibility that MUC1-C induces ALDH1 activity and thereby mammosphere formation. However, silencing ALDH1A1 in MCF-7/MUC1-C cells had no apparent effect on mammosphere formation, indicating that MUC1-C confers mammosphere formation by an alternative mechanism (Fig. 7G). To provide further support for involvement of MUC1-C in self-renewal activity, we silenced MUC1-C in HER2+ SKBR3 and triple-negative MDA-MB-468 cells. Here again, downregulation of MUC1-C significantly decreased the capacity of these breast cancer cells to form mammospheres. Silencing MUC1-C in MDA-MB-468 cells is also associated with suppression of ALDH1 activity [23]. However, consistent with findings in MCF-7/MUC1-C cells, silencing ALDH1A1 in MDA-MB-468 cells had little if any effect on mammosphere formation, indicating that increases in ALDH1A1 activity are dispensable for anchorage-independent growth (Fig. 7G). Other work has shown that MUC1-C induces EMT in breast cancer cells by activating the ZEB1/miR-200c regulatory loop [25]. In this context, EMT increases the capacity of mammary epithelial cells to form mammospheres [8], providing an alternative mechanism by which MUC1-C could promote non-adherent growth and survival (Fig. 7G).

MUC1-C contributes to activation of the canonical NF-κB pathway [31]. MUC1-C also interacts directly with NF-κB p65 and promotes activation of NF-κB-dependent genes [24]. Other work has linked NF-κB activation to mammosphere formation and breast cancer-initiating activity [11-13, 34]. In this respect, we found that inhibition of MUC1-C-induced NF-κB activity is associated with suppression of self-renewal mammosphere formation, indicating that MUC1-C induces anchorage-independent growth by an NF-κB-mediated pathway. The available evidence supports involvement of NF-κB in conferring stemness and sphere formation by inducing IL-8 signaling [35, 36, 42]. Indeed, consistent with induction of the IL-8 pathway, MUC1-C increased NF-κB occupancy of the IL-8 promoter and inhibition of MUC1-C resulted in downregulation of IL-8 expression. The proximal promoter of the *IL-8* gene has adjacent NF-κB- and C/EBP-binding sites that cooperate in transactivation [42, 43]. In addition, NF-κB and C/EBP physically form complexes on target gene promoters and cooperate in the activation of *IL-8* transcription [44]. C/EBPβ plays an important role in mammary gland development by regulating stem cell repopulating activity [45]. Indeed, recent studies have shown that MUC1-C interacts with C/EBPβ, occupies the ALDH1A1 promoter with C/EBPβ and thereby contributes to upregulation of ALDH1A1 expression [23]. Therefore, the present studies do not exclude the possibility that MUC1-C may further enhance IL-8 transcription by promoting interaction between NF-κB p65 and C/EBPβ on the IL-8 promoter. Consistent with MUC1-C-induced activation of NF-κB and the presence of a functional NF-κB binding site on the CXCR1 promoter, we also found that MUC1-C drives CXCR1 expression by an NF-κB-dependent mechanism. These findings support a pathway in which MUC1-C activates NF-κB and induces IL-8 and CXCR1 expression, which in turn drives self-renewal capacity and mammosphere formation (Fig. 7G). Thus, in this model, targeting MUC1-C is sufficient to suppress the IL-8/CXCR1 signaling pathway and decrease self-renewal. Other work has shown that that IL-6 can contribute to MCF-7 mammosphere formation [46] and IL-8-mediated survival [43]. Accordingly, additional studies will be needed to determine whether MUC1-C also contributes to the regulation of IL-6 expression in breast cancer cells.

MUC1-C forms complexes with HER2/HER3 at the breast cancer cell membrane and contributes to their activation [19, 20]. The MUC1-C cytoplasmic domain contains a CQC motif that is necessary and sufficient for the formation of MUC1-C homodimers, which are of importance for MUC1-C function [19]. Thus, targeting MUC1-C with silencing or by blocking the CQC motif in HER2+ breast cancer cells results in inhibition of p-HER2 activation and the downstream AKT pathway [20]. HER2 drives stem cell-like activity by AKT→β catenin signaling [47, 48] and through an interaction between HER2 and CXCR1/2 [18]. Therefore, the present finding that targeting MUC1-C in HER2+ SKBR3 cells inhibits mammosphere formation could be a consequence of p-HER2 downregulation. Nonetheless, we also found that silencing MUC1-C suppresses the formation of triple-negative MDA-MB-468 mammospheres. In addition, expression of the MUC1-C(CQC→AQA) mutant blocked MDA-MB-468 sphere formation, indicating that the MUC1-C CQC motif and MUC1-C homodimerization are necessary for anchorage-independent growth. The formation of MUC1-C homodimers is also necessary for import of MUC1-C into the nucleus, where it interacts with transcription factors, such as NF-κB [19]. Thus, targeting MUC1-C homodimerization by treatment with inhibitors, such as GO-203, blocks MUC1-C-induced activation of the NF-κB pathway [24], and thereby induction of IL-8/CXCR1 expression and mammosphere formation. Previous studies with other cell-penetrating peptides that bind to the MUC1-C CQC motif demonstrated that blocking MUC1-C homodimerization is effective in killing breast cancer cells growing in vitro and as tumor xenografts [49]. The present work extends these observations by demonstrating that silencing MUC1-C in MDA-MB-468 cells markedly inhibits tumorigenicity. These effects of targeting MUC1-C are associated with increases in reactive oxygen species (ROS) and the induction of late apoptosis/necrosis [49]. By extension, MUC1-C protects cells from increases in ROS associated with exposure to hypoxia and glucose deprivation [19]. Increasing evidence indicates that cancer stem-like cells maintain low ROS levels and that disruption of ROS defense mechanisms results in loss of their survival [50-53]. In this way, dependence of breast cancer cell survival on activation of NF-κB, and thereby cytokine signaling that can further activate NF-κB in a positive feedback loop, may contribute to maintenance of ROS levels and self-renewal. Therefore, based on the present findings, targeting MUC1-C in breast cancer-initiating cells with downregulation of NF-κB activation could in turn disrupt redox balance and induce loss of self-renewal.

Finally, a Phase I trial of GO-203 has been completed in patients with refractory solid tumors and, as a result, definition of a maximum tolerated dose for Phase II studies. The finding that targeting MUC1-C with GO-203 inhibits self-renewal of breast cancer stem-like cells supports further evaluation of GO-203 alone and in combination with other agents used for the treatment of breast cancer. In this way, GO-203 has been synergistically combined with tamoxifen [40], trastuzumab [20] and the cytotoxic drugs, taxol and doxorubicin [54], and therefore could be used clinically with these agents to more effectively treat the breast cancer stem-like, initiating cell population.

## METHODS

### Cell culture

Human MCF-7 and MDA-MB-468 breast cancer cells were cultured in DMEM with 10% heat-inactivated fetal bovine serum (FBS), 100 units/ml penicillin, 100 μg/ml streptomycin, and 2 mM L-glutamine. SKBR3 breast cancer cells were grown in McCoy's 5A medium containing FBS, antibiotics and glutamine. MCF-7, MDA-MB-468 and SKBR3 cells were transduced with a lentiviral vector expressing a MUC1 shRNA (Sigma), a NF-κB p65 shRNA (Sigma) or, as a control, with a scrambled shRNA vector (CshRNA) as described [23]. MCF-7 cells were stably transfected with a control pHR-CMV vector or one expressing MUC1-C [23]. Cells were treated with the MUC1-C inhibitor GO-203, the control CP-2 [20] or the NF-κB pathway inhibitor BAY11-7085 (Santa Cruz Biotechnology).

### Immunoprecipitation and immunoblotting

Whole cell lysates were prepared in NP-40 lysis buffer and analyzed by immunoblotting with anti-MUC1-C (LabVision), anti-NF-κB p65 (Santa Cruz Biotechnology) and anti-β-actin (Sigma) as described [25]. Immune complexes were detected using horseradish peroxidase-conjugated secondary antibodies and enhanced chemiluminescence (GE Healthcare).

### Quantitative RT-PCR

For qRT-PCR analysis, cDNA synthesis was performed with 1 μg total RNA using the Thermoscript RT-PCR system (Invitrogen). cDNA samples were amplified using the SYBR green qPCR assay kit (Applied Biosystems) and the ABI Prism 7000 Sequence Detector (Applied Biosystems). Primers used for qRT-PCR detection of IL-8 and CXCR1 mRNAs are listed in Supplemental Table S1. Statistical significance was determined by the Student's t-test.

### NF-κB transcriptional activity

Cells (5 × 10^**5**^) growing in six-well plates were transfected in the presence of Superfect transfection reagent (Qiagen) with (i) 1 μg of the p-NF-κB-Luc plasmid containing NF-κB-activated sequences upstream to the luciferase reporter (pGL4.32/luc2P/NF-κB-RE/Hygro; Promega) and (ii) 1 ng of a control reporter plasmid (pRL-TK) containing the *Renilla* gene under control of the TK promoter. After 48 h, the cells were harvested and lysed in passive lysis buffer. Luciferase activity was analyzed using the Dual Luciferase Assay System (Promega). Relative luciferase activity is reported as the fold-induction after normalization for transfection efficiency.

### Measurement of ALDH activity

The Aldefluor assay kit (Stem Cell Technologies) was used for determination of ALDH enzymatic activity. Cells were suspended in aldefluor assay buffer and incubated with the ALDH enzyme substrate, BODIPY-aminoacetaldehyde (BAAA), for 40 min at 37°C. As a control, cells were also treated with diethylaminobenzaldehyde (DEAB), an inhibitor of ALDH enzyme activity. Fluorescence was determined using a BD Biosciences LSRFortessa flow cytometer and analyzed using FACSDiva software (BD Biosciences). Statistical significance was determined by the Student's t-test.

### Mammosphere culture

Single-cell suspensions were cultured in MammoCult™ Human Medium Kit (Stem Cell Technologies) at a density of 2,000 to 10,000 cells per well of a 6-well ultralow attachment culture plate (Corning CoStar). For first generation M1 culturing, cells were grown with replenishment of the medium twice over 7 days. For second M2 generation culturing, M1 mammospheres were harvested, incubated with trypsin for 3 min at 37°C, and mechanically dispersed by gentle pipetting. Single cells were confirmed under a microscope, counted and resuspended in fresh MammoCult™ medium. Mammospheres were visualized using a Nikon inverted TE2000 microscope and scored as positive when ≥50 μm in size. Sphere forming efficiency (SFE) was calculated by dividing the number of mammospheres by the number of suspended cells.

### Chromatin immunoprecipitation (ChIP) assays

Soluble chromatin was prepared from 2-3 × 10^**6**^ cells as described [23] and precipitated with anti-NF-κB or a control nonimmune IgG. For re-ChIP assays, NF-κB complexes from the primary ChIP were eluted and reimmunoprecipitated with anti-MUC1-C as described [23]. The SYBR green qPCR kit was used for ChIP qPCRs with the ABI Prism 7000 Sequence Detector (Applied Biosystems). Relative fold enrichment was calculated as described [55]. Primers used for qPCR of the IL-8 promoter and control region are listed in Supplemental Table S2.

### Assessment of tumorigenicity

MDA-MB-468/CshRNA and MDA-MB-468/MUC1shRNA cells growing in log-phase cultures were trypsinized and washed twice with sterile PBS. Viable cells were determined by trypan blue exclusion. Cells (4 × 10^**6**^) suspended in 0.2 ml sterile PBS were injected subcutaneously into the flanks of 4-6 week old female BALB/c nu/nu mice. Tumor volumes were calculated using the formula V=(L × W^**2**^)/2, where L and W are the larger and smaller diameters, respectively.

## SUPPLEMENTARY FIGURES AND TABLES


