# Supplementary material for: Targeting the MUC1-C oncoprotein inhibits self-renewal capacity of breast cancer cells
Source: Oncotarget. 2014 Mar 24;5(9):2622–34. doi: 10.18632/oncotarget.1848 (PMC4058032; doi:10.18632/oncotarget.1848)
Supplement: Supplementary file 1 [file oncotarget-05-2622-s001.doc]

**Supplemental Tables**

**Supplemental Table S1: qRT-PCR primers for IL-8 and CXCR1.**

| IL-8-Fwd | 5’-ACATACTCCAAACCTTTCCACCC-3’ |
| --- | --- |
| IL-8-Rev | 5’-CAACCCTCTGCACCCAGTTTTC-3’ |
| CXCR1-Fwd | 5’-TGCATCAGTGTGGACCGTTA-3’ |
| CXCR1-Rev | 5’-TGTCATTTCCCAGGACCTCA-3’ |
| GAPDH-Fwd | 5’-CCATGGAGAAGGCTGGGG-3’ |
| GAPDH-Rev | 5’-CAAAGTTGTCATGGATGACC-3’ |

**Supplemental Table S2: ChIP primers.**

| NBR-Fwd | 5’-TCGTCATACTCCGTATTTGATAAGG-3’ |
| --- | --- |
| NBR-Rev | 5’-CGATTTGCAACTGATGGCC-3’ |
| CR-Fwd | 5’-AGTGGCATGATAACAGCTCC-3’ |
| CR-Rev | 5’-TAGTCCCAGCTACTCAGGAG-3’ |

NBR: NF-κB binding region. CR: control region

**
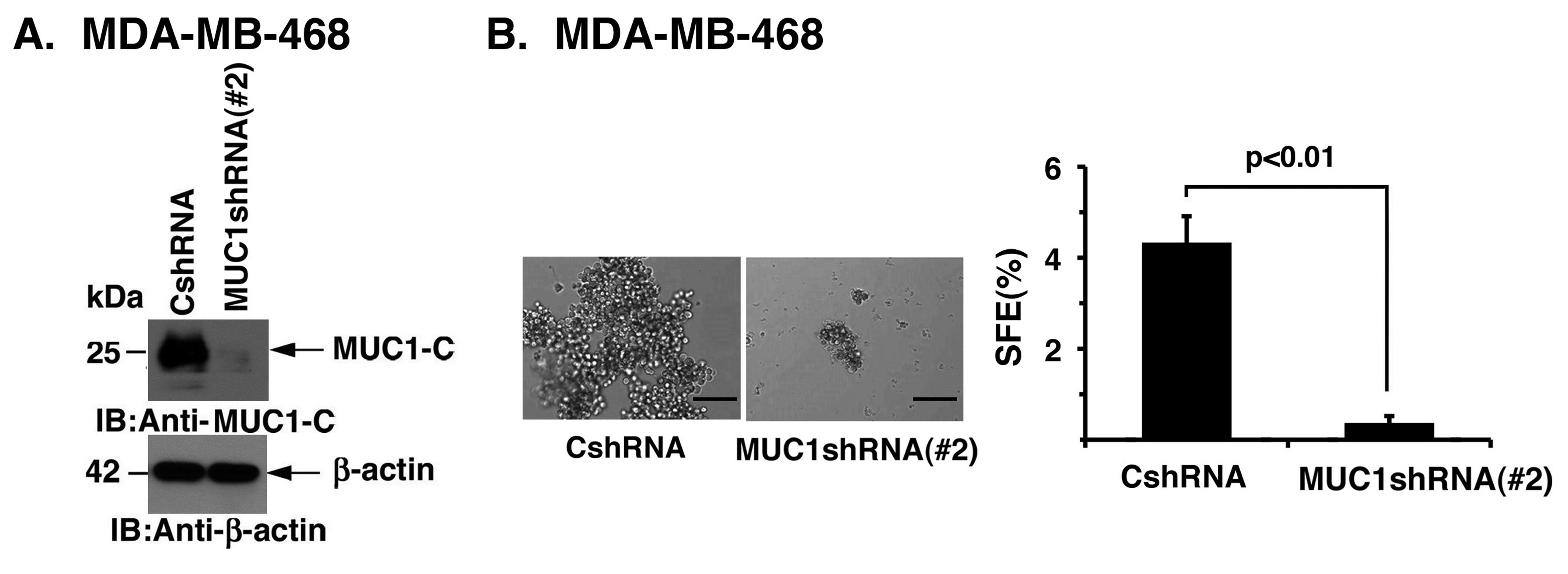
**

**Supplemental Figure S1.** A. MDA-MB-468 cells were infected with lentiviruses stably expressing the CshRNA or MUC1shRNA(#2). Lysates of MDA-MB-468/CshRNA and MDA-MB-468/MUC1shRNA(#2) cells were immunoblotted with the indicated antibodies. B. Representative images are shown of MDA-MB-468/CshRNA and MDA-MB-468/MUC1shRNA(#2) cells grown in mammosphere culture medium (left). Bar represents 100 microns. The percentage SFE is expressed as the mean±SD of three determinations (right).


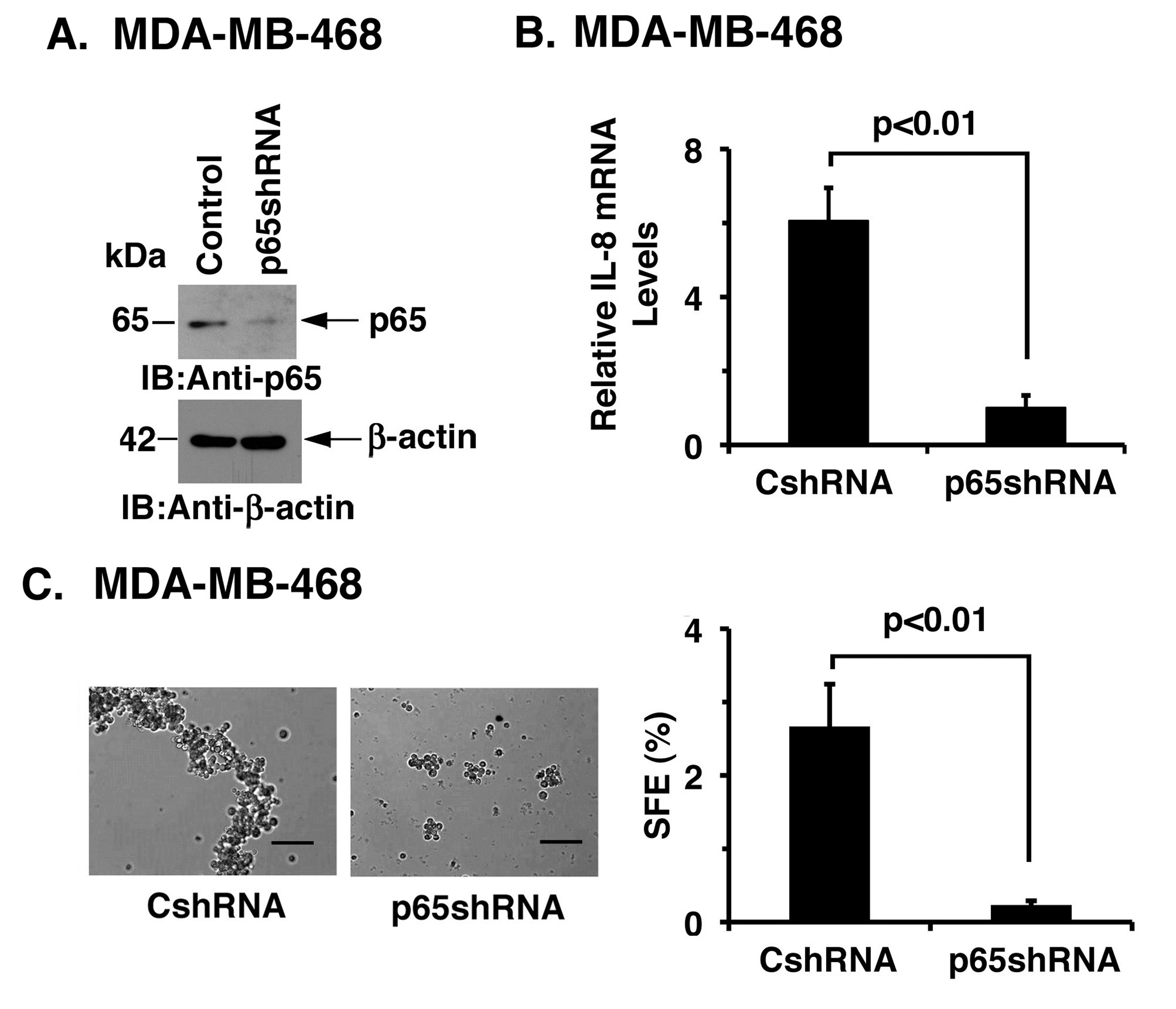


**Supplemental Figure S2.** A. Lysates from MDA-MB-468 cells expressing a control CshRNA or a p65shRNA were immunoblotted with the indicated antibodies. B. MDA-MB-468/CshRNA and MDA-MB-468/p65shRNA cells were analyzed for IL-8 mRNA levels by qRT-PCR. The results are expressed as relative mRNA levels (mean±SD of three determinations) as compared with that obtained for GAPDH (p65shRNA cells assigned a value of 1). C. MDA-MB-468/CshRNA and MDA-MB-468/p65shRNA cells were seeded in mammosphere culture medium for 5 d. Images are shown for the indicated cells (left). Bar represents 100 microns. The percentage SFE is expressed as the mean±SD of three determinations (right).


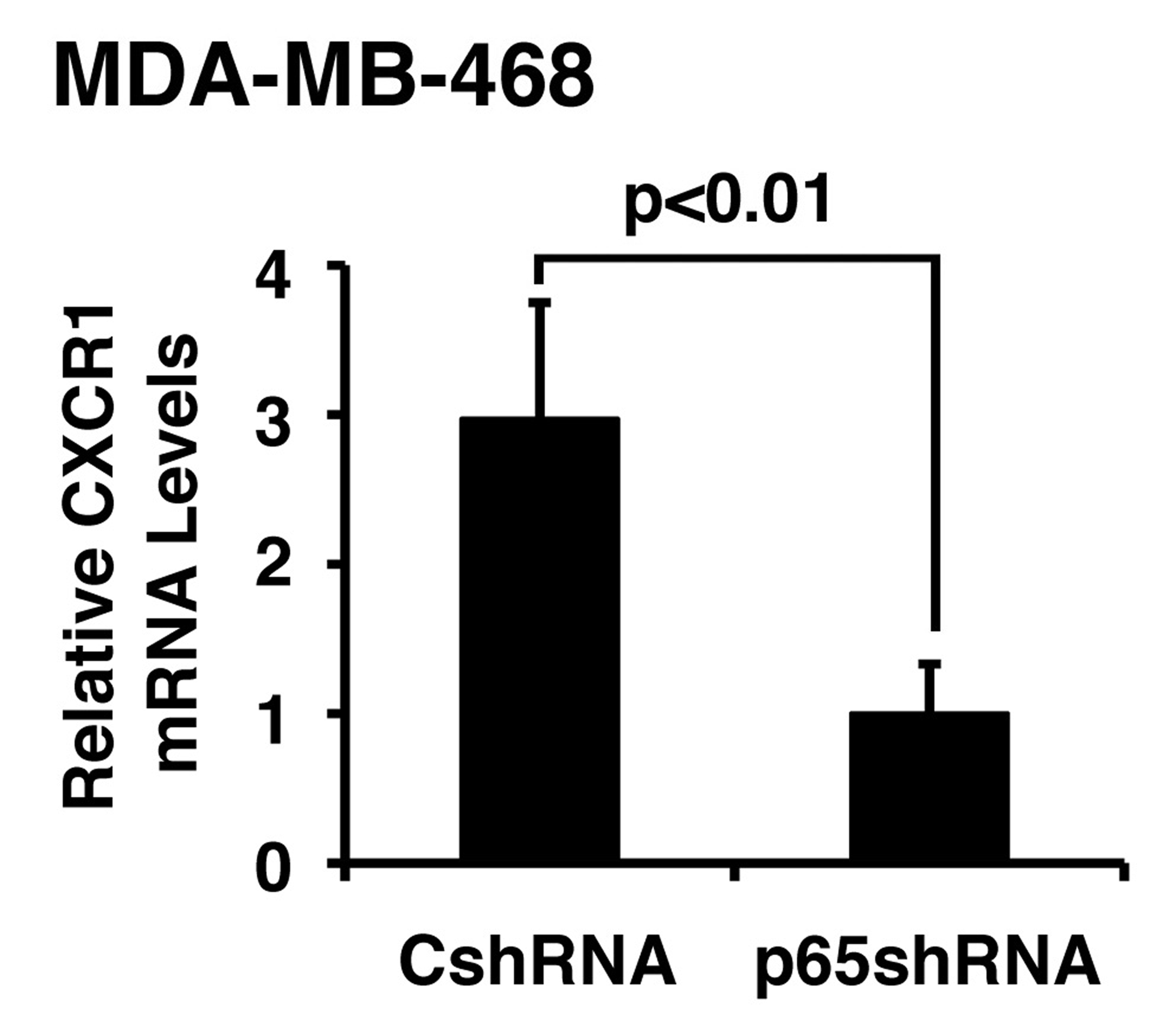


**Supplemental Figure S3.** MDA-MB-468/CshRNA and MDA-MB-468/p65shRNA cells were analyzed for CXCR1 mRNA levels by qRT-PCR. The results are expressed as relative mRNA levels (mean±SD of three determinations) as compared with that obtained for GAPDH (p65shRNA cells assigned a value of 1).
